# Sustainable resource management with bone char—challenges and opportunities for enhancing soil health and phosphorus stocks

**DOI:** 10.1007/s42773-025-00550-3

**Published:** 2026-02-28

**Authors:** Majid Ghorbani, Nazanin Azarnejad, Robert W. Brown, David R. Chadwick, Stefano Loppi, Davey L. Jones

**Affiliations:** 1 Department of Life Sciences (DSV), University of Siena, 53100 Siena, Italy; 2 School of Environmental and Natural Sciences, Bangor University, Bangor, Gwynedd LL57 2UW UK; 3 BioAgry Lab, Department of Life Sciences (DSV), University of Siena, 53100 Siena, Italy; 4 NBFC, National Biodiversity Future Center, 90133 Palermo, Italy

**Keywords:** Circular economy, Phosphorus availability, Resource management, Soil fertility, Sustainable agriculture

## Abstract

The global annual production of animal by-product (ABP)-derived bone, estimated at 95‒126 million tonnes, presents both an environmental challenge and an opportunity for sustainable resource utilization. We estimate that bone char (BC) could theoretically replace 13‒32% of the global phosphorus (P) fertilizer market. BC, produced through the pyrolysis of animal bones, has emerged as a promising material for use in a range of agricultural applications related to soil fertility and water quality. The conversion of ABP-derived bone into BC through pyrolysis not only eliminates potential human and animal pathogens (e.g., prions, viruses, bacteria), but also creates a valuable resource rich in P, calcium, and magnesium. This review synthesizes current research on the potential applications of BC in agriculture, focusing on its multifunctional role as a slow-release P fertilizer, a carbon (C) storage material, and an effective adsorbent for remediating contaminated soils. Field and laboratory studies demonstrate that BC’s performance is strongly influenced by pyrolysis conditions, with optimal temperatures between 300 and 500 °C for nutrient release applications and above 600–800 °C for enhanced surface area and contaminant remediation. Its hydroxyapatite structure enables gradual P release and potential toxic element (PTE) immobilization, while its porous nature can provide new habitat niches for soil microorganisms and improve soil water retention. In comparison to most conventional inorganic fertilisers, BC can enhance soil fertility by releasing P slowly, thereby improving plant growth and productivity, particularly in acidic soils. The low cost, renewable nature, and ease of regeneration of BC further enhance its appeal as a viable solution for mitigating environmental pollution and promoting sustainable resource management practices. Beyond its established applications, this review identifies critical knowledge gaps, including the need to investigate BC’s long-term impacts on soil health, microbial communities, and greenhouse gas emissions. We also discuss opportunities for optimizing production methods and expanding applications beyond agriculture. Given BC’s potential to address multiple agricultural and environmental challenges, we emphasize the importance of interdisciplinary research to evaluate implementation barriers, including economic viability, social acceptance, and regulatory frameworks.

**Graphical Abstract:**

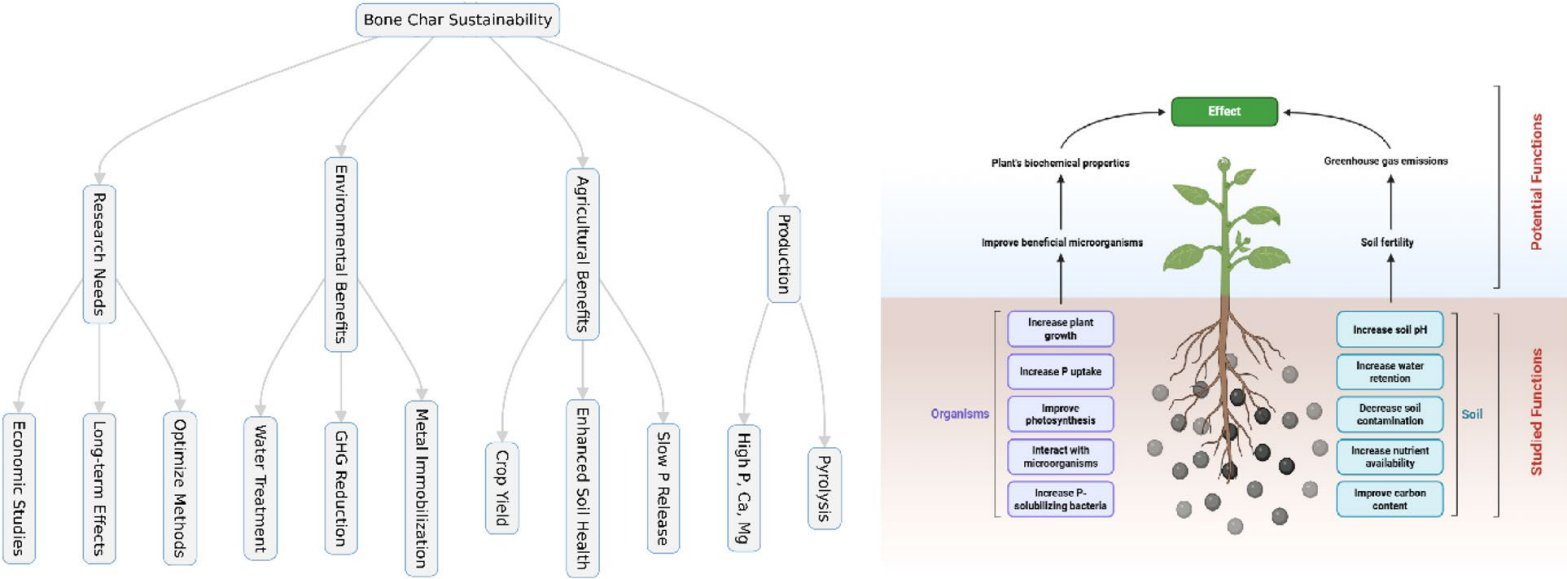

## Highlights

- 95‒125 million tonnes of waste bone feedstock are created annually to produce bone char (BC).
- BC could theoretically replace 13‒32% of the global phosphorus (P) fertilizer market.
- BC enhances soil fertility through slow P release and contaminant removal.
- BC improves soil microbial activity, water retention and remediation of PTEs.
- Long-term effects of BC on soil health and greenhouse gas emissions require further research.

## Introduction

Global population growth and intensifying human activities are degrading environmental resources, undermining the delivery of many critical ecosystem services (Jafari et al. 2022). Agricultural intensification has generally led to reductions in soil health and nutrient stocks, increasing the need for synthetic fertilizers and pesticides to maintain or increase crop yields (Jones et al. 2013). Nitrogen (N) and phosphorus (P) deficiencies have been particularly problematic, as these elements are crucial to ensure healthy plant growth and maintain yields in food production. In particular, P is an essential but finite resource, primarily sourced from mined rock phosphate and utilized in mineral fertilizers (Scholz et al. 2013). Additionally, nutrient retention in soils is increasingly an issue, with synthetic fertilizer application leading to the loss of nutrients via erosion, runoff, leaching and gaseous emissions (including volatilisation) (e.g., PO_4_^3−^, NO_3_^−^, N_2_O, NH_3_,), resulting in eutrophication and atmospheric pollution (Jones et al. 2013; Rehana et al. 2022). Additionally, soils store significant amounts of carbon (C) (Batjes 2014), which should be protected from degradation and ideally increased in order to help remove (or offset) greenhouse gas emissions (GHG) contributing to climate change (Kumar et al. 2020; Longbottom et al. 2022). Increasing the sustainability and efficiency of resource use is a key aim of sustainable agricultural intensification. In the context of P, recycling high volume, P-rich animal by-products (ABPs) from industrial activities as fertilizers is one way of increasing resource use efficiency (e.g., meat and bone meal, wastewater treatment biosolids, steel slag; Solangi et al. 2023).

Bones typically constitute 15‒20% of the weight of domesticated animals (i.e., poultry, cattle goat, and pigs). This proportion varies significantly between species, with mature cattle having the highest bone content relative to body mass. Based on global meat production (approx. 350 million t y^−1^; FAO 2023) and a dressing percent of 47% for cattle, 50% for sheep, and 70% for pigs (Moreira et al. 2021; AHDB 2024), we estimate that this generates approximately 95‒126 million tonnes of slaughterhouse ABP-derived bone and associated processing residues each year (Ghosh et al. 2016; Nassar et al. 2021; Sharma et al. 2022; FAO 2023). Overall, the biggest global producers of ABP-derived bone are China, Brazil, and the USA, which together account for approx. 50% of global production (FAO 2023). Further, with the projected 15‒20% growth in global meat protein availability by 2050, ABP-derived bone generation is expected to significantly increase in the coming decades (Warner 2019; OECD-FAO 2022; Liang et al. 2023). Utilizing bone by-product material has potential as an abundant, low-cost, and nutrient dense resource. However, following the ban on using meat and bone meal (MBM) for cattle feed in the EU due to the bovine spongiform encephalopathy crisis (Commission Decision 94/381/EC), there is a significant amount of animal waste requiring safe disposal or transformation. The ban highlighted the need for alternative processing methods that could ensure pathogen inactivation while preserving valuable nutrients. Modern thermochemical conversion technologies, operating under strict regulatory frameworks, now offer pathways to safely transform these materials into high-value BC products. Developing technology to convert waste materials into value-added products can address this challenge. For example, thermochemical conversion of ABP-derived bones can be used to reduce the overall volume of waste, while producing porous materials that offer environmental benefits (Sierra et al. 2023). Currently, abattoir waste is categorized based on risk, with high-risk materials (e.g., specified risk materials (SRMs); carrying transmissible spongiform encephalopathies (TSEs), blood, and offal) requiring incineration, while low risk materials can be utilized with restrictions after specific treatment for purposes such as agricultural fertilizer (Darch et al. 2019; EU Regulation 2019), as well as animal feed (Bharadwaj et al. 2002), water purification (Shahid et al. 2019), or energy production (Kefalew and Lami 2021). Once these materials have passed through designated regulatory “end points” and appropriate processing stages, they may be considered for further transformation applications, including thermochemical conversion. However, we note that direct use of fresh abattoir waste for biochar production is not permitted under current EU regulations. It should be noted that animal by-product regulations vary significantly across countries, with some jurisdictions potentially allowing direct thermochemical conversion of raw ABP-derived bone materials (Category 2–3) under appropriate safety protocols and high-temperature processing conditions (> 500 °C) that ensure complete pathogen and prion (> 600 °C) inactivation. The conversion of processed animal-derived materials (such as rendered bone meal) into biochar through pyrolysis offers a promising pathway for resource recovery, provided strict regulatory compliance and pathogen inactivation protocols are followed regardless of the specific regulatory framework. ABPs and their derived products in the EU are regulated under Regulation (EC) No 1069/2009 and Commission Regulation (EU) No 142/2011; EU-marked fertilising products must comply with Regulation (EU) 2019/1009. Bone-derived materials used as fertilisers must originate from eligible ABP categories and reach an ABP end point before use in EU fertiliser products (EU Regulation 2019). Pyrolysis/gasification then falls under CMC 14 requirements, with Annex I limits (e.g., metals, hygiene) applying at product level. These frameworks, along with operator preference for high-temperature processing (mostly ≥ 850 °C for water-treatment-grade char), dominate current industrial practice and adoption (EU Regulation 2019).

The production and addition of biochar to agricultural soils have been proposed as a potential way to promote soil organic carbon (SOC) storage and the delivery of other ecosystem services (Piccirillo 2023). Biochar is produced by the pyrolysis of organic materials (feedstock) under low oxygen conditions at temperatures >350 °C, with the resulting material often exhibiting a high C content exceeding 70% (Enaime et al. 2020). Feedstocks can be sourced from a wide range of materials, from virgin wood and biomass to more complex industrial residues and byproducts, with their use determined by applicable regulations (Wang et al. 2022). As such, the thermochemical conversion of bone material into biochar offers multiple benefits, namely, the elimination of biological contaminants including bacteria (e.g., *Escherichia coli*, *Salmonella* spp., *Campylobacter* spp.), viruses (e.g., Bovine Viral Diarrhea Virus, Foot and Mouth Disease), and prions (e.g., BSE, CJD, scrapie) (Zaharioiu et al. 2022). The pyrolysis process also concentrates and transforms P into slow-release forms and creates a stable C structure (Ducey et al. 2017; Bayata and Mulatu 2024).

Animal bones, and thus bone chars (BC), are primarily composed of Ca (20‒25%), P (10‒12%), and Mg (0.5‒1%), with lesser amounts of Na, K and micronutrients also present. Consequently, a range of investigations have been undertaken exploring the potential of using BC to enhance P recycling and replace conventional rock P-derived fertilizers (Warren et al. 2009; Someus and Pugliese 2018). The P present in BC is predominantly bound to Ca (e.g., hydroxyapataite, β-tricalcium phosphate, tetracalcium phosphate) and is therefore bioavailable to plants (Stávková and Marousek 2021). Additionally, BC has a unique mesoporous structure and surface charge distribution, offering distinct advantages for adsorption compared to other biochars (Li et al. 2023). Pyrolyzed BC, however, does share some similar physicochemical properties with conventional biochar, offering long-term C stability in soil, albeit BC is relatively low in C (8‒15% by weight) compared to other wood and crop residue based-chars (40‒90% C by weight). BC also tends to be much richer in Ca, P, and Mg relative to conventional biochar (Table 1; Liu et al. 2020). Although its P is primarily embedded within the char structure, making it less immediately available than conventional fertilizers, the slow release of nutrients through gradual dissolution contributes to enhanced soil fertility, agricultural productivity, and soil contaminant immobilization (Siebers and Leinweber 2013; Frišták et al. 2019; Azeem et al. 2022). This dual function, C sequestration and nutrient provision, depends on several factors such as pyrolysis temperature and residence time, which can be optimized to balance stability with nutrient availability (Zimmer et al. 2018; Piccirillo 2023). BC’s potential as an alternative and renewable, slow-release P fertilizer can contribute to efforts in recycling P for more sustainable agricultural practices (Sun et al. 2018). In addition, soil contamination through cadmium (Cd) and uranium (U) may occur following the application of some conventional fertilizers, representing a serious environmental challenge (Amann et al. 2018).

**Table 1:** Comparison of the properties of typical wood-based biochar and bone char

Property	Wood-based biochar	Bone char
Production temperature	400 °C–800 °C	600 °C to 1000 °C ≥ 850 °C (industrial use)
Feedstock	Wood (e.g., hardwood, softwood)	C2 and C3 animal bones (e.g., bovine, ovine, porcine)^a^
Carbon content	60‒80%	10‒25%
Surface area	200‒600 m^2^ g^−1^	150‒300 m^2^ g^−1^
Porosity	High (60‒90%)	High
pH	9‒11	7‒9
Ash content	10‒30%	30‒90%
Bulk density	0.2‒0.6 g cm^−3^	0.5‒1.2 g cm^−3^
Electrical conductivity	Low to moderate (0.1‒2 mS cm^−1^)	Moderate to high (2‒10 mS cm^−1^)
Nutrient content	Lower in nutrients (except K)	High in Ca and P and some trace elements
P content	Low (1‒5 g kg^−1^)	High (75‒140 g kg^−1^)
P_2_O_5_ content	0.1–1%	17–36%
Cation exchange capacity	Moderate (100‒2000 meq kg^−1^)	High (30‒1200 meq kg^−1^)
Anion sorption capacity	Moderate (10‒100 mg kg^−1^)	High (50‒200 mg kg^−1^)
Water holding capacity	High (150‒300% of DW)	Moderate (50‒150% of DW)
Carbon stability	Stable	Stable
Adsorption properties	Adsorbs organic compounds, potential toxic elements (PTEs)	Adsorbs PTEs, phosphate, and organic compounds

^a^C2 and C3 refer to categories of animal by-products (ABPs) based on their potential health risks. Category 2 (C2) refers to high-risk materials, while Category 3 (C3) is classified as low-risk materials

Here we estimate the amounts of conventional P fertilizer that could be averted from using BC in place of rock P. Firstly, we assumed that the volume of ABP-derived bone generated globally was 95‒125 × 10^6 ^t y^−1^ and that conversion of this waste stream through pyrolysis (yielding approx. 30‒40% of the original bone mass as char) could theoretically produce 28.5‒50 million tonnes of BC annually. With a P content typically ranging from 8‒14% in BC (Table 1), this translates to approx. 2.1‒7.0 million tonnes of P potentially available for fertilizer applications. When compared to the global P fertilizer consumption of 20‒22 × 10^6^ t y^−1^ (McDowell et al. 2024), BC could theoretically replace 13‒32% of the global P fertilizer market, representing a significant potential contribution to global P resources. Additionally, BC may offer agronomic advantages over conventional P fertilizers (see below). The implementation of large-scale BC production would therefore help create a circular economy solution for this waste stream. The practical realization of this potential, however, depends on several factors including production efficiency, P bioavailability in diverse agricultural systems, and the economic feasibility of establishing collection and processing infrastructure at scale.

The potential application of BC in sustainable agriculture is evident, yet the absence of a comprehensive review of its agricultural applications is apparent. Hence, the aim of this review is to synthesize previous studies on BC application focusing on: (i) production, (ii) comparison to conventional P fertilizers, (iii) societal, economic and policy considerations, (iv) environmental safety, and finally (v) to identify research gaps.

## Methodology

**Fig. 1:**
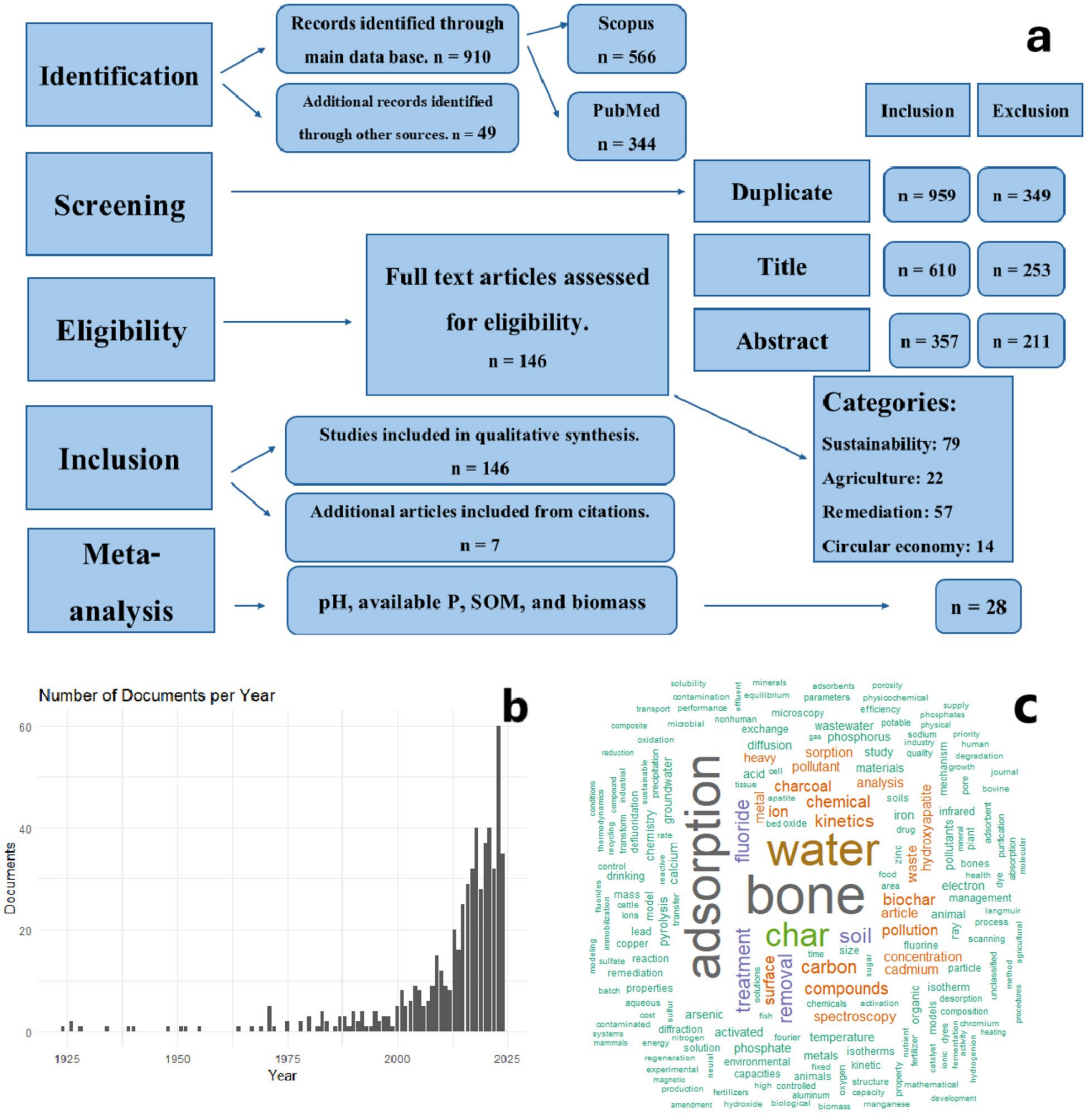
PRISMA flow diagram visually representing the screening and selection process (**a**). Number of articles on bone char published since 1924 to 2024 (**b**). Word cloud of records’ keywords (**c**)

This systematic review synthesizes primary data from academic and industrial publications on the application of BC in agriculture and its environmental impact, following the PRISMA (Preferred Reporting Items for Systematic Reviews and Meta-Analyses) guidelines. A comprehensive search was conducted using keyword queries in Scopus and PubMed, incorporating terms such as “bone char,” “bone-based biochar,” “bone-derived biochar,” “biochar from bone,” and “bone meal” (Fig. 1). The search covered literature published between 1924 and 2024, limited to English-language publications (Fig. 1). Additional relevant literature was identified from databases including ScienceDirect, Google Scholar, and other scholarly repositories by direct search. The screening and selection process initially identified 959 papers and chapters. After removing 349 duplicates, 610 unique sources remained for further screening. During the title screening, 253 sources were excluded as they lacked specific evidence on BC or were deemed out of scope (e.g., studies on wood-based biochar, non-agricultural applications, or theoretical modelling papers). An additional 211 sources were removed after the abstract screening, leaving 146 for full-text assessment. Of these, 22 were review articles, and 124 were primary research papers. Additionally, 7 more articles were included from citation tracking (Fig. 1). Studies were manually selected at this stage based on their relevance to existing or novel BC classifications and documented environmental impacts, which included positive, neutral, or negative effects, as well as some other criteria, e.g., having control and BC treatments and consisting of at least three replicates per treatment. The review adheres to the PRISMA framework, ensuring a transparent and systematic approach to the identification, screening, eligibility, and inclusion of studies.

A meta-analysis evaluated the effect of BC on selected soil and crop parameters (e.g. soil pH, organic matter, available P, and plant biomass) using the response ratio (RR), which was calculated as the natural logarithm of the ratio between the mean value for the BC treatment and the control. The 95% confidence intervals (CI) were calculated alongside standard error (SE) values. Data were extracted on soil pH, available P, soil organic matter (SOM), and plant biomass, including control and BC treatment means, standard deviations, sample sizes, application rates, soil types, crop types, and experiment durations (Fig. 1). Studies missing these values were excluded, resulting in 28 published sources being taken forward for use in the meta-analysis.

Forest plots were generated to illustrate response ratios and confidence intervals using R packages including dplyr and ggplot2 (Wickham 2016; Wickham et al. 2020) in the R environment (v4.3.2; R Core Team, 2023). Sensitivity analysis excluded studies with small sample sizes or high variability to test result robustness. Key limitations which were identified in the meta-analysis included study heterogeneity (soil type, crop type, application rate), reporting bias, and variability in measurement methods.

## Results and discussion

### BC production and resulting characteristics

**Fig. 2:**
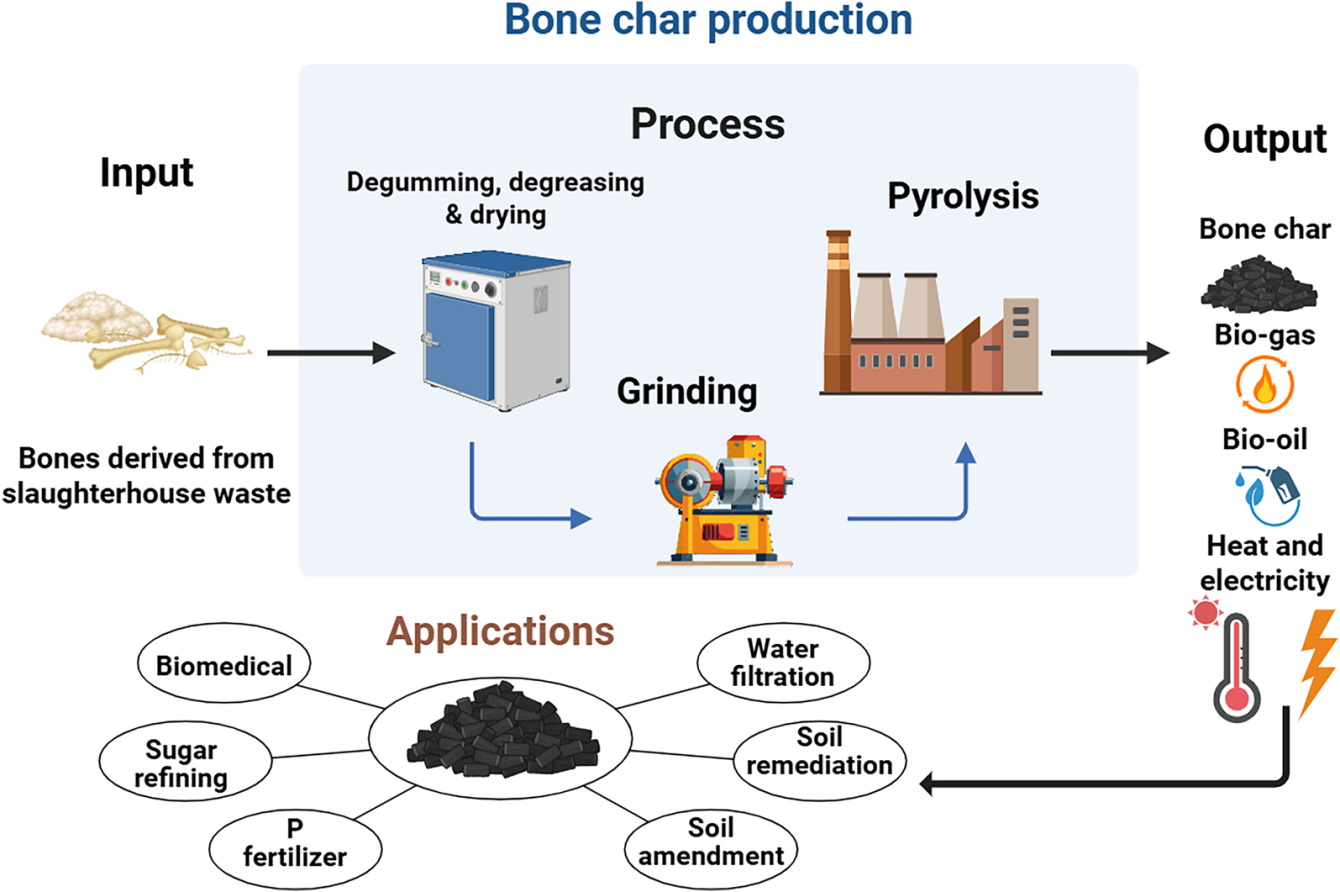
Schematic diagram showing the bone char (BC) production process and its potential applications

Fresh animal bone typically consists of approximately 65‒70% minerals (primarily hydroxyapatite), 20‒25% organic matter (mainly type I collagen protein), 5‒8% water, and 2‒5% fat, with trace amounts of non-collagenous proteins, blood proteins, and polysaccharides also present (Field et al. 1974; Doyle 1979; Olszta et al. 2007). The transformation of these materials during pyrolysis (Fig. 2) and the physicochemical properties of the resulting BC are highly dependent on temperature, heating rate, and residence time (Leinweber et al. 2018). These properties are further influenced by feedstock characteristics (e.g., bone type, particle size, moisture content) and atmosphere (Rojas-Mayorga et al. 2015; Rafiq et al. 2016), although specific operating conditions can be tailored to optimize yields of different products such as biochar, syngas, or bio-oil (Panwar et al. 2012; Irfan et al. 2016). Prior to pyrolysis, the raw materials must undergo preparation, which typically includes degumming, degreasing, drying, and crushing (Li et al. 2024). Unlike other types of biochar which can be produced by low temperature torrefaction (Lin et al. 2023), pyrolysis of bone typically requires temperatures >350 °C to initiate effective thermal transformation, with temperatures commonly ranging from 500 to 600 °C for agricultural applications, though industrial bone char production for water purification employs much higher temperatures (> 850 °C) (Table 1). The choice of pyrolysis temperature involves important trade-offs: while higher temperatures (> 600 °C) enhance hydroxyapatite crystallinity, reduce porosity by collapsing the organic matrix, and increases its ash content due to the loss of organic matter (Figueiredo et al. 2010; Cantrell et al. 2012; Zimmer et al. 2018; Li et al. 2023), they also increase energy consumption, reduce C retention, and may decrease P bioavailability due to the formation of more stable phosphate phases (Piccirillo 2023). The enhanced crystallinity aids in reducing the solubility of the calcium phosphate (Ca-P) minerals while also raising the pH through greater metal oxide formation, particularly CaO, with smaller amounts of K_2_O and Na_2_O (Siebers and Leinweber 2013; Leinweber et al. 2019; Ahmed et al. 2021; Amin 2023). The higher pH is also partially due to BC having a lower C content than conventional biochar and thus fewer phenolic and carboxylic groups.

Animal bones and BC are predominantly composed of hydroxyapatite (Ca_10_(PO_4_)_6_(OH_2_), as confirmed by X-ray diffraction and X-ray near edge fine structure analysis (Medellin-Castillo et al. 2014; Rojas-Mayorga et al. 2015; Zwetsloot et al. 2015, 2016; Robinson et al. 2018). The elemental composition and atomic ratios of BC, particularly the Ca/P ratio, can be influenced by the pyrolysis temperature and residence time (Hart et al. 2022). At low temperatures (200‒500 °C), hydroxyapatite remains the dominant phase with initial calcium carbonate (CaCO_3_) decomposition and minor β-tricalcium phosphate formation (Ca_3_(PO_4_)_2_, 5‒10%), while at higher temperatures (600‒1000 °C), significant mineral transformations occur including hydroxyapatite dehydroxylation, increased β-tricalcium phosphate formation (up to 35%), emergence of tetracalcium phosphate (Ca_4_(PO_4_)_2_O) and calcium pyrophosphate (Ca_2_P_2_O_7_), and enhanced crystallinity leading ultimately to sintering (Zwetsloot et al. 2015; Patel et al. 2015; Hart et al. 2022). The sequence of transformations greatly affects the properties of BC, with mineralogical changes occurring most rapidly between 500 and 700 °C where the dominant hydroxyapatite phase undergoes significant modification through dehydroxylation and partial conversion to other Ca-P phases (Rojas-Mayorga et al. 2013). Additionally, as pyrolysis temperature and residence time increase, both porosity and specific surface area of BC decrease due to microstructural transformations, with the extent of these changes influenced by the original mineral composition and animal origin of the bones (Rajendran et al. 2013; Patel et al. 2015). Adjusting the solubility and bioavailability of P in BCs to match crop nutrient requirements can therefore be achieved through appropriate pyrolysis temperatures (Piccolla et al. 2021).

**Fig. 3:**
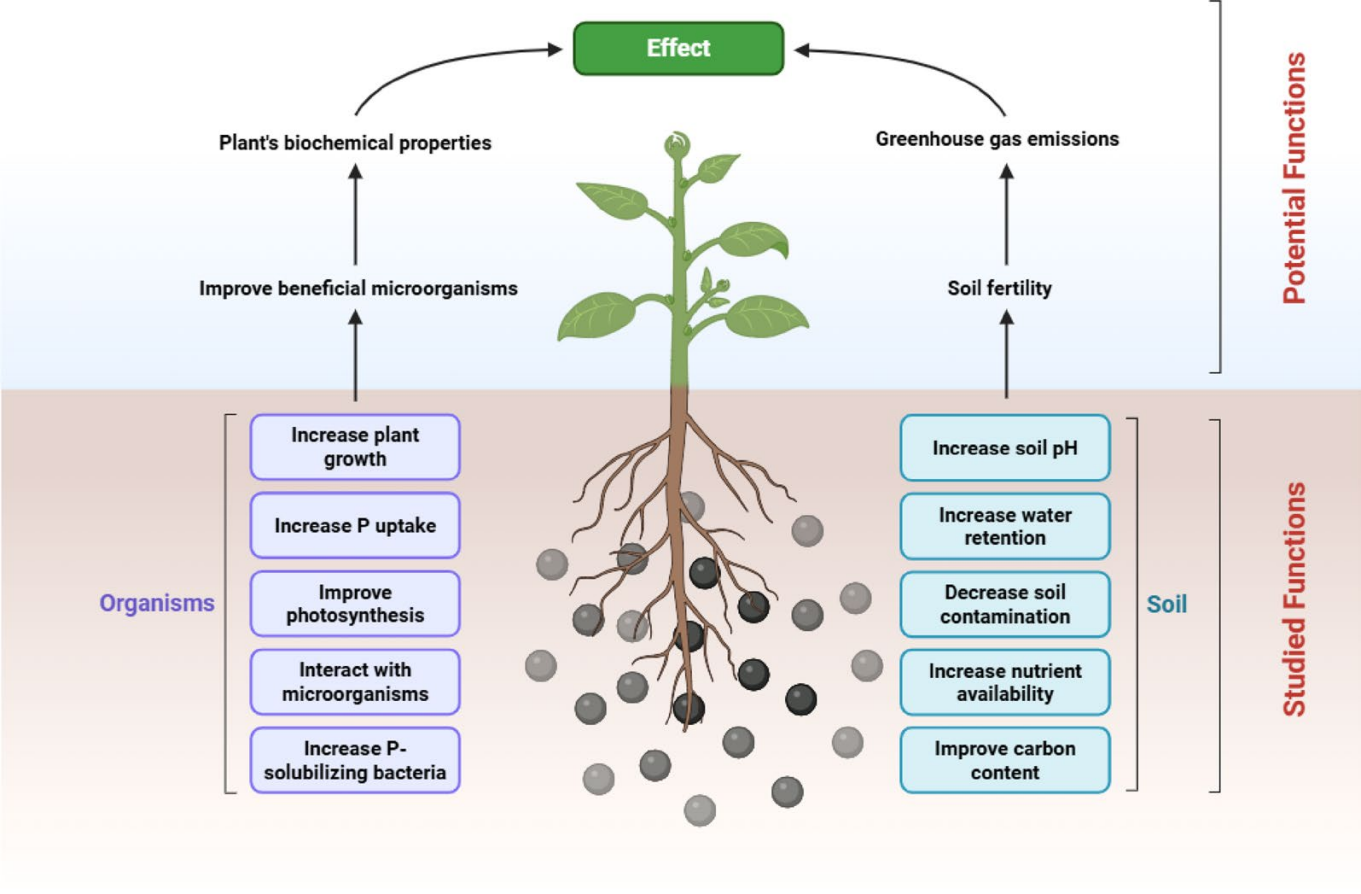
Schematic representation of the studied and potential functions of bone char in agriculture

Sodium bicarbonate extractions (Olsen P) have often been used to assess P availability in waste materials (Christiansen et al. 2020). Using this approach, studies have shown that cattle bone-derived BC has total P values ranging from 75 to 140 g kg^−1^ depending on pyrolysis temperature, but that the available P represented only a small fraction of this P pool (150‒1500 mg kg^−1^), albeit still much greater than present in most agricultural soils (2‒50 mg kg^−1^) or in wood-based chars (1‒100 mg kg^−1^) (Warren et al. 2009; Amin 2020, 2023). Based on the solubility of hydroxyapatite (solubility product constant (*K*_sp_) = 2.9 × 10^−58^), β-tricalcium phosphate (*K*_sp_ ≈ 10^−29^) (Samavedi et al. 2013), tetracalcium phosphate (*K*_sp_ ≈ 10^−34^) and calcium pyrophosphate (*K*_sp_ ≈ 10^−17^), P solubility should increase with pyrolysis temperature, however, this response is not always apparent with more available P present at lower pyrolysis temperatures (Ahmed et al. 2021; Amin 2023). We ascribe this apparent contradiction between theoretical solubility constants and observed P availability to multiple competing factors beyond simple mineral phase transitions including (i) surface area dynamics and porosity changes, particularly that lower temperature pyrolysis often produces char with higher surface area and porosity, (ii) residual organic matter effects, specifically that more organic matter remains at low pyrolysis temperatures, which can form complexes with Ca^2+^, preventing Ca binding strongly to P, and (iii) local pH microenvironments (i.e. higher pyrolysis temperatures increase metal oxide formation and alkalinity that can inhibit P dissolution). Besides its P fertilizing effects, BC can offer multiple benefits as soil amendments, by improving soil quality (e.g., raising pH) and reducing nutrient and pesticide leaching into groundwater (Table 1; Fig. 3; Mendes et al. 2019; Alkharabsheh et al. 2021; Bolan et al. 2022).

In summary, BC production through pyrolysis involves complex interactions between feedstock characteristics and pyrolysis conditions, resulting in diverse physicochemical properties. Understanding these factors is essential for optimizing BC production processes and harnessing its potential applications in various fields, including agriculture and environmental remediation. Based on our synthesis of the available evidence, it is clear that further research is needed to explore the effects of different pyrolysis parameters on BC properties and its performance in practical applications. For example, potential research areas may include: (1) optimizing two-stage pyrolysis protocols where initial low-temperature treatment (400‒500 °C) is used to preserve surface functionality followed by targeted high-temperature exposure (700‒800 °C) to create engineered bone chars with both high P availability and beneficial structural properties; (2) investigating the theoretical and practical possibilities of the co-pyrolysis of bone with complementary biomass (e.g., legume residues) to produce designer chars with balanced nutrient profiles and C sequestration potential; and (3) exploring post-pyrolysis treatments such as acid washing or steam activation to enhance P availability or surface properties without compromising structural integrity.

### BC application to agricultural soils

#### BC as a soil amendment

It is currently estimated that 40% of agricultural land has been degraded due to human activities (UNCCD, 2022; D’Odorico and Ravi 2023). As the demand for food intensifies and soils become increasingly depleted, BC has the potential to partially address the global land degradation challenge by helping to restore soil quality and a range of UN Sustainable Development Goals (SDGs; Lamb et al. 2005; Lal 2015). With nutrient depletion affecting over 30% of the world’s agricultural soils, particularly through P deficiency (MacDonald et al. 2011), BC application to land directly contributes to SDG 2 (Zero Hunger) by enhancing soil fertility, increasing crop productivity, and improving nutrient retention. Beyond nutrient management, BC also demonstrates the ability to help ameliorate soil contamination, due to its ability to immobilize PTEs, pesticides and xenobiotics (Hussain et al. 2022; Mei et al. 2022). These functions align closely with SDG 6 (Clean Water and Sanitation) through reducing groundwater contamination and filtering water systems (Sorlini et al. 2011; Fung et al. 2021), while simultaneously advancing SDG 13 (Climate Action) by sequestering C and potentially reducing GHGs from agricultural practices (Saleh et al. 2020). The circular economy approach of BC production also supports SDG 12 (Responsible Consumption and Production) by transforming potentially contaminated animal byproducts into valuable soil amendments (Hart et al. 2022). Moreover, these materials contribute to SDG 15 (Life on Land) by potentially restoring soil health, preventing degradation, and supporting biodiversity.

**Fig. 4:**
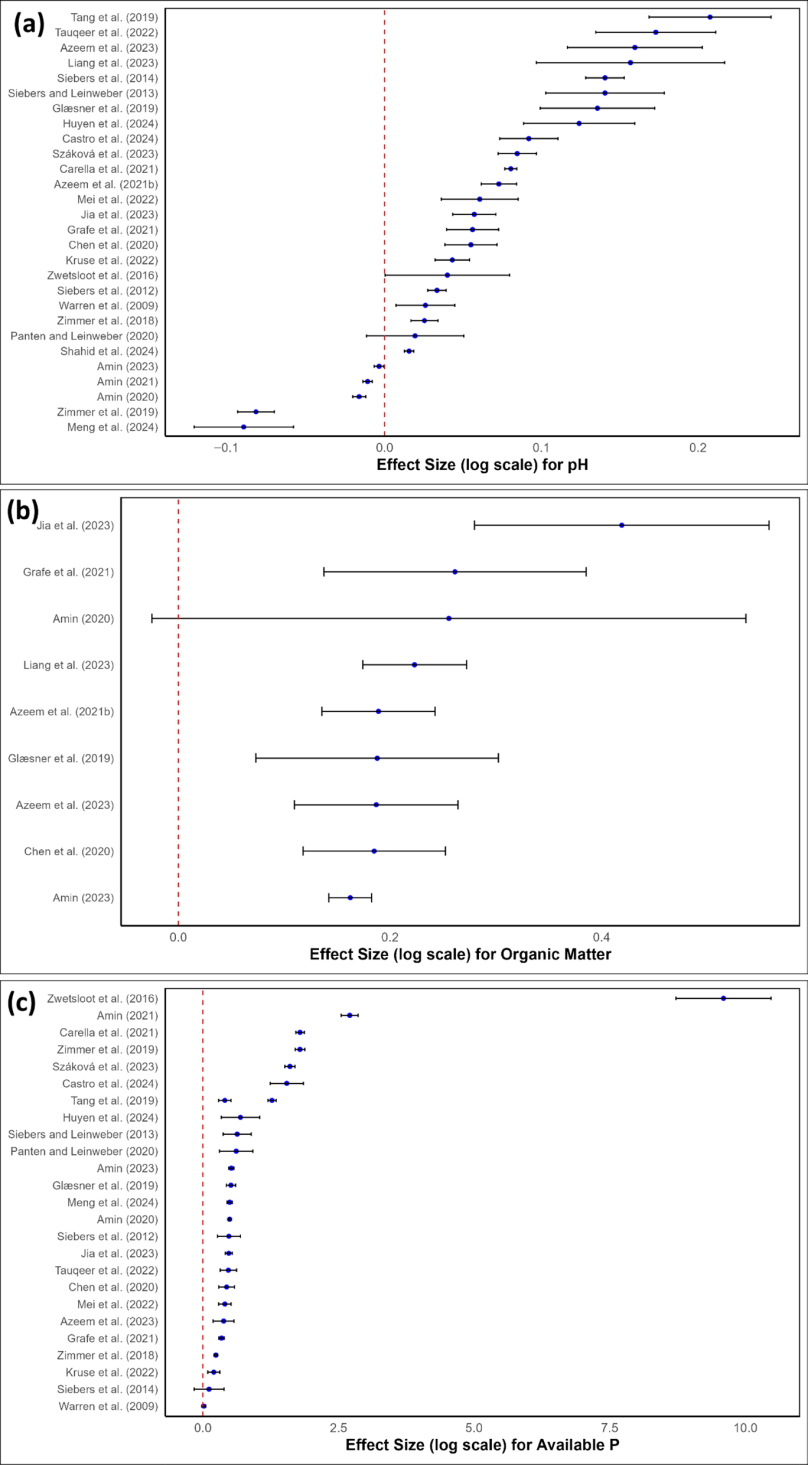

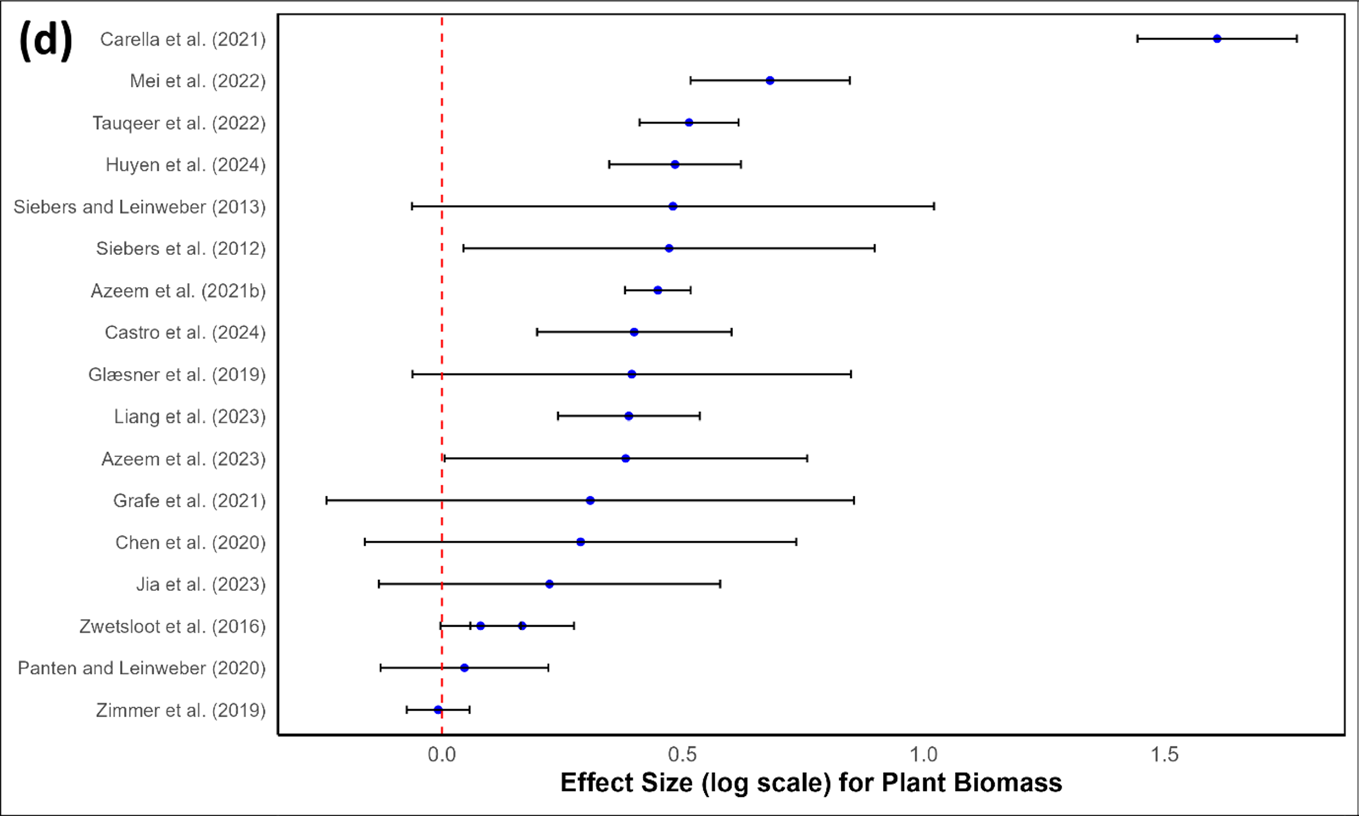
Forest plots showing the response ratios (RR) and 95% confidence intervals (CI) for bone char (BC) application across four key parameters: **a** soil pH, **b** soil organic matter (SOM), **c** available P, and **d** plant biomass. The vertical dashed line represents no effect (RR = 0), with points to the right indicating a positive effect and points to the left indicating a negative effect of BC compared to the control

Similar to wood-derived biochar, BC has also emerged as a versatile tool for remediating soils contaminated with PTEs. The ability of BC to immobilize heavy metals can be ascribed to its highly porous structure and high Ca-P content, which create an abundance of binding sites for contaminant capture through ion exchange, and surface complexation and precipitation mechanisms (Karppinen et al. 2017; Mendes et al. 2019; Azeem et al. 2021b, 2022; Liu et al. 2021). The remediation efficacy of BC has been demonstrated across multiple PTEs, including Pb, Cd, Zn, and Cr (VI). When applied to contaminated soils, BC can also reduce metal bioavailability by increasing soil pH (Fig. 4a), transforming metals into less mobile minerals (Zendehdel et al. 2017; Shen et al. 2018a, b). For example, studies with Chinese cabbage showed marked reductions in plant Pb uptake (Chen et al. 2006), while research on Cd mobility demonstrated decreased uptake by arable crops (Siebers and Leinweber 2013; Siebers et al. 2014; Morshedizad et al. 2016). Practical applications have also shown promising results across various crops. Research using sheep and chicken bone-derived BC in maize (*Zea mays*) cultivation demonstrated reduced metal concentrations in both soil and plants, alongside improved root and shoot development (Azeem et al. 2021a, b). Similar benefits were observed in pea (*Pisus sativus*), where BC application led to reduced foliar Cu, Zn, Pb, and Cd concentrations while enhancing growth (Mei et al. 2022). Recent research has also shown the benefits of combining BC with other materials, such as clay composites, showing enhanced efficacy in Cd immobilization in *Brassica rapa* (Li et al. 2018). Additionally, BC has demonstrated potential in removing organic contaminants, including herbicides (Mendes et al. 2019) and petroleum hydrocarbons (Karppinen et al. 2017) from soil, though this area warrants further investigation. Remediation applications typically require BC production at temperatures above 600 °C to reduce metal bioavailability or maximize contaminant removal (Rojas-Mayorga et al. 2016; Gruden et al. 2017; Shen et al. 2018a, b; Wang et al. 2018; Amin 2023). We ascribe this to higher temperatures leading to an increased surface area, enhanced aromaticity, reduced functional groups, greater hydrophobicity, higher metal oxide content, and a greater potential to raise pH.

Beyond metal remediation, BC offers additional soil benefits. It has been shown to significantly enhance water retention capacity, particularly in sandy soils, with studies showing increased water holding capacity from 1.3% (control) to up to 5.5% with 10% BC addition (Leinweber 2017). While BC contains less C than traditional biochar (i.e., derived from wood or agricultural residues), it shares similar beneficial effects on soil properties, including enhanced cation exchange capacity and pH modification (Table 2; Fig. 4a, b; Glaser et al. 2001; Atkinson et al. 2010; Hagemann et al. 2017). The meta-analysis showed that BC significantly increased soil pH across different soil types (Fig. 4a). The response ratio for pH varied across studies, with the greatest increase observed in acidic soils or higher application rates. This suggests that BC application may effectively reduce soil acidity, making it a promising amendment for acidic agricultural systems. However, some studies reported minor or no changes, likely due to differences in BC pyrolysis temperature and soil buffering capacity (e.g. Zimmer et al. 2019; Amin 2021; Meng et al. 2024).

**Table 2:** Summary of the studies conducted on the effects of bone char (BC) application on soil, plant, and microorganisms. NA, not applicable

Feedstock	Pyrolysis	Experiment (soil, species, etc.)	Dose	Key finding	References
Cattle bones	400 °C, 45 min	BC, phosphate rock & triple superphosphate on soil available P	NA	BC is a better source of P in comparison with the others	Warren et al. (2009)
Pig bones	800 °C, 1 h	P solubilizing bacteria	NA	60% of isolates showed positive scores; they belonged to the genera *Arthrobacter*, *Bacillus*, *Burkholderia*, *Collimonas*, *Paenibacillus*, *Pseudomonas*, *Serratia*, and *Streptomyces*	Postma et al. (2010)
Mixed bones	NA	Onion, potato and wheat	1–2 g P kg^−1^, granules < 5 mm	Increase potato yield, decrease onion yield, no effect on wheat	Siebers et al. (2012)
Mixed bones	700–800 °C	Cd contaminated soils	0, 100, 500, and 1000 mg P kg^−1^	The P dissolution from BC was negatively correlated with pH and positively with P sorption capacity. BC as sustainable P fertilizer and useful soil amendment	Siebers and Leinweber (2013)
Bovine bones	> 800 °C	Soil elemental composition	45 kg P ha^−1^,	P content increased in soil and increased the plant availability of BC-derived nutrients	Zimmer et al. (2018)
Pig bone and wood biochar	650 °C, 2 h	Soil, soil enzymatic activity, plant growth and nutrient uptake	0, 0.5%, 1%, 2%, and 4% (w/w)	Increase CEC, EC, pH and SOC. Increase soil and plant K, P and N. Improve plant growth and soil enzymatic activity	Chen et al. (2020)
Sheep bones	500–800 °C	Soil quality, maize growth and phytoavailability of Cd and Zn	0, 2%, 5% and 10% (w/w)	Increase SOC, total N and P. Decrease Zn and Cd in maize roots and shoot. Plant yield increased	Azeem et al. (2021b)
Bones (not specified)	> 800 °C	Winter wheat, abundance of microorganisms	NA	High abundance of bacteria, increasing P availability influenced by bacterial P turnover, and increasing P content of the plant	Grafe et al. (2021)
Pork bones		Pea growth and PTE contamination		Enhance pea growth and reduce metal accumulation. Decrease the leachability of PTEs	Mei et al. (2022)
Cow bones	500 and 800 °C	Soil, microbial biomass, bacterial community, maize growth	0, 2.5%, 5% and 10% (w/w)	Enhanced total nutrient content and nutrient availability. Higher microbial biomass, especially in 500 °C	Azeem et al. (2023)
Cow bones	800 °C	Soil properties and P pools	5-year incubated BC	The treatment effects were mostly insignificant for P pools. Sulfur modification of BC can improve P availability of BC at the field scale	Jia et al. (2023)
Pork bones	400 and 600 °C	Soil, rice and microorganisms	Micro & nano BC, 5 and 25 g kg^−1^	MNBCs significantly increased the Cd-treated rice biomass by up to 25.0%. MNBCs significantly reduced the acid-soluble fraction and increased the residual fraction of Cd. MNBCs greatly reduced the Cd distribution at the subcellular level. High dose of 600 °C MNBCs reduced the Cd content in rice by up to 73.51%. MNBCs improved the microbial diversity and abundance in the Cd-amended rhizosphere	Liang et al. (2023)
Male pig bones	100, 200, and 300 °C for 12 h	Soil and microorganisms	NA	Elevation of phosphate-solubilizing fungi (PSF) abundance. Increase P mobility in the soil. The bacterial community showed fewer sensitive responses to the BC addition	Meng et al. (2024)
Cow bones	1000 °C	Soil, maize and GHG emissions	1% (w/w)	BC increased P content and plant growth in low P soil and decreased GHG emissions and global warming potential	Ghorbani et al. (2025)

The effects of BC on SOM (Fig. 4b) were generally positive, with a moderate increase observed across studies. The meta-analysis suggested that BC contributes to SOM accumulation by increasing soil carbon storage. However, the variability in the response ratios suggests that the effect depends on soil type and BC application rate. Some studies showed limited effects, possibly due to differences in the initial SOM levels or variations in BC composition. While BC may provide some soil C input (Lehmann et al. 2011; Ghorbani et al. 2025), its low organic C content (Table 1), especially when produced at high pyrolysis temperatures, limits its potential for significant C sequestration compared to other high-C biochar materials. The climate benefits of BC are more likely derived from its role in circular P economy and reduced environmental impacts of phosphate mining rather than direct C storage. Further research is needed to properly assess these indirect climate benefits alongside any C sequestration effects.

#### BC as a P-rich fertilizer

P is a critical macronutrient for plant growth, yet its low bioavailability in soil often limits optimal plant development. With traditional rock phosphate mining facing sustainability challenges, BC has emerged as a promising alternative P source (Simons et al. 2014). The effectiveness of BC as a P fertilizer is controlled to a large extent by pyrolysis conditions and soil chemistry. Pyrolysis temperature critically influences P solubilization properties (Etok et al. 2007; Reyes-Gasga et al. 2008; Li et al. 2015), with distinct optimal ranges for different applications. For P fertilization, temperatures between 300–400 °C have proven most effective (Biswas et al. 2021; Ahmed et al. 2021; Piccolla et al. 2021; Soja et al. 2023). Soil pH plays a crucial role in P availability from BC. In acidic soils, enhanced apatite dissolution increases P bioavailability, while neutral or alkaline conditions can restrict P release (Warren et al. 2009; Morshedizad et al. 2016; Leinweber et al. 2018; Zimmer et al. 2018; Glaser and Lehr 2019; de Castro et al. 2026). This pH-dependent behaviour highlights the importance of understanding local soil conditions for optimal BC application. BC offers several advantages as a P fertilizer, particularly in contaminated soils. Pot experiments have demonstrated BC’s superior performance compared to traditional P fertilizers in Cd-contaminated soils with adequate P levels (Siebers et al. 2014). The material’s slow-release characteristics minimize nutrient leaching while ensuring sustained P availability (Warren et al. 2009; Morshedizad et al. 2018). Furthermore, BC’s properties can be optimized through controlled production conditions and modification techniques to enhance P solubility and overall effectiveness (Siebers and Leinweber 2013; Zimmer et al. 2018; Chen et al. 2020; Azeem et al. 2021b, 2023; Mei et al. 2022). From a sustainability perspective, BC represents a circular economy approach by converting ABP-derived bones into a valuable fertilizer (Siebers and Leinweber 2013; Vassilev et al. 2013; Amin 2020). While current P release rates from BC may not match conventional chemical fertilizers (Siebers et al. 2012, 2014; Zimmer et al. 2018), its potential for sustainable long-term P management is promising (Meng et al. 2024).

The meta-analysis showed a significant increase in available P, especially in P-deficient soils (Fig. 4c). However, the magnitude of the increase varied generally depending on application rate and soil type. The highest response ratios were observed in calcareous and acidic soils (e.g. Zwetsloot et al. 2016; Amin 2021), where BC improved P availability through slow-release mechanisms. The results of the studies conducted to study the effect of BC on soil available P are presented in Fig. 4c and Table 2. The benefits of bone char (BC) are demonstrated by Ghorbani et al. (2025), who found that BC application greatly enhanced P availability for maize growth across different soil types, increasing P levels up to 1000-fold in low-P podzolic brown soils (from < 0.1 to ~ 100 mg kg^−1^) and more than 100-fold in moderate-P orthic brown soils (from < 1.5 to ~ 150 mg kg^−1^).

#### Effects on nutrient cycling and crop productivity

BC, being rich in P, plays a crucial role in enhancing nutrient availability and cycling in soil ecosystems (Fig. 3). The presence of BC in soil ecosystems can stimulate the proliferation of beneficial soil microorganisms (e.g. phosphate- or sulphur-solubilizing bacteria), which are essential for nutrient cycling, soil fertility maintenance, and overall ecosystem health (Qian et al. 2015; Azeem et al. 2022; Castillo et al. 2023).

The addition of BC to soil increases nutrient availability (e.g., P and Ca) and improves a range of soil physiochemical properties, which in turn gives rise to several co-benefits including enhanced plant growth, pigmentation and antioxidant production (Ali et al. 2017). For example, a significant increase in plant growth in response to BC addition has been reported for maize (Azeem et al. 2021b), pak choi (*Brassica chinensis*; Chen et al. 2020), and fenugreek (*Aspergillus niger*; Tauqeer et al. 2022). It is likely that this response is highly dependent on the grain size and amount of BC added to soil, however, information on this aspect remains limited. The high surface area of BC (150‒300 m^2^ g^−1^) may also aid in directly and indirectly promote nitrogen (N) retention in soil. For example, the negatively charged surface of BC can sorb NH_4_^+^, reducing its diffusion and potential transformation to NO_3_^−^, thus leading to less gaseous emissions of both NH_3_ and N_2_O (Hagemann et al. 2017). Morshedizad and Leinweber (2017) also reported that BC enhances soil physical conditions, particularly beneficial for crops grown in sandy soils prone to water stress. Moreover, the general porous structure of BC serves as a habitat for various microorganisms, with non-pathogenic fungi successfully colonizing BC, utilizing the P supply from the char (Postma et al. 2013; Morshedizad and Leinweber 2017). While the porous structure of BC (e.g., pore volume and pore sizes) is strongly dependent on pyrolysis temperature and residence time (Alkurdi et al. 2020), this physical characteristic provides a habitat for beneficial microorganisms while potentially restricting pathogen movement by sorbing chemotaxis signalling molecules used in pathogen host location. This has led to studies investigating BC’s potential for biological control, which to date have shown promising results for managing soil-borne pathogens in agricultural substrates (Castillo et al. 2023), albeit this research remains in its infancy.

Limited research exists on the effects of BC on crop yields in the field, primarily stemming from the focus of most research on short-term pot experiments. Siebers et al. (2012) observed yield increases for potatoes, no significant effects on wheat, and yield decreases for onions compared to control groups without P addition. Similarly, Siebers et al. (2014) found that while wheat yield responded positively to BC application, lettuce and potato tuber yields showed inconsistent results across soils with varying P levels. In another study, Little et al. (2015) investigated the impact of BC on the growth of weeds and arable crops in comparison to composted poultry manure in biological farming systems. They concluded that BC did not adequately replicate the P supply provided by composted poultry manure. Contrary findings were reported by Zwetsloot et al. (2016), who observed positive effects of BC in combination with arbuscular mycorrhizae (AM) inoculation on maize yield in a P-fixing soil. Maize inoculated with AM showed similar P accumulation whether fertilized with BC (pyrolyzed at 750 °C) or triple superphosphate (TSP).

In summary, the fertilization effect of BC varies considerably between studies, sometimes yielding positive outcomes but not consistently across all studies (Fig. 4d, Table 2). While the meta-analysis showed an overall positive trend, some studies showed high variability, which is reflected in their confidence intervals (Fig. 4d). The greatest improvements were observed in P-deficient soils where BC increased nutrient availability (e.g. Carella et al. 2021; Mei et al. 2022). Ghorbani et al. (2025) reported that BC led to substantial increases in maize height and biomass in low-P soils, with improvements up to 4-fold higher compared to the unamended control and soil amended with plant-derived biochar. In contrast, in moderate-P soils, where P is not a limiting factor, no significant differences in plant biomass were observed during the early stages of the experiment. However, BC effectiveness can vary, as some studies in some cases report no significant changes in plant biomass, suggesting that other limiting factors (e.g., N availability, soil pH or crop-specific responses, may influence BC performance (e.g. Zimmer et al. 2019; Panten and Leinweber 2020). Further research is therefore required to better understand the potential effectiveness of BC on nutrient cycling and crop productivity, particularly at the field scale and in direct comparison to conventional biochar and other sources of inorganic and organic P. Given the slower nutrient-release properties of BC and the apparent variable response in different soil types, these should also be multi-year, multi-site trials that include combined treatments of BC and e.g. TSP, recognising that BC may not always be able to supply sufficient P to meet crop demand.

#### Interaction of BC with microorganisms

Microorganisms are pivotal in the transformation of soil P into forms accessible to plants (Richardson et al. 2009). Phosphate-solubilizing bacteria (PSB) are common in soils (constituting 0.5‒50% of total bacterial populations; Vazquez et al. 2000; Gyaneshwar et al. 2002), and have been shown to enhance the solubility of P-containing minerals by secreting low molecular weight organic acids, such as gluconic acid, citric acid, and oxalic acid (Zhu et al. 2018), increasing soil P concentrations up to ∼ 1000 times (Wu et al. 2013; Mendes et al. 2015; Li et al. 2016). These organic acids complex cations (e.g., Ca, Fe, Al) bound to phosphate, liberating H^+^ protons and inducing P solubilization (Kpomblekoua and Tabatabai 1994). BCs produced at high temperatures have been applied in conjunction with PSB to mitigate pathogen risks (Postma et al. 2010, 2013). However, increased crystallinity and decreased porosity of bone apatite at temperatures beyond 500 °C could diminish P release efficiency (Etok et al. 2007; Figueiredo et al. 2010). Achieving a balance between pyrolysis temperature and P release dynamics of BCs with PSB addition is therefore crucial, highlighting the urgent need to undertake fundamental research to optimize BC functionality prior to field deployment.

The utilization of selected microorganisms with specific functionalities is another avenue explored in advanced and sustainable agriculture (Das et al. 2022). While a comprehensive analysis of this topic is beyond the scope of this review, we discuss studies involving BC where microorganisms were employed (Table 2). Zwetsloot et al. (2016) investigated BC in combination with root hairs and AM fungi. This combination yielded promising results in maize pot experiments, with comparable P accumulation and dry mass increase to traditional TSP fertilization. This study highlights the potential of fully exploiting BC as a fertilizer, warranting further field-scale investigations.

Another promising approach involves combining BC with PSB. As indicated above, these strains solubilize hydroxyapatite-P through organic acid production, which may promote plant growth (Santana et al. 2019; Li et al. 2020). BC is also likely to generate a “charosphere” (Quilliam et al. 2013) similar to that of biochar, leading to its potential use as a carrier for functional inoculants for biocontrol. For example, Postma et al. (2013) demonstrated the effectiveness of strains of the genera *Pseudomonas* in the presence of BC in suppressing *Pythium* and *Fusarium* species, pathogens responsible for damping off, and crown and root rot in tomato plants. Based on the inherent properties of BC (Table 1), current evidence suggests that BC could serve as an excellent carrier for delivering biocontrol bacteria into soil or substrate, offering a dual benefit of biocontrol and recycling of P-rich waste products. In a recent study, a composite of carboxymethyl cellulose, BC, and FeS was prepared for PSB immobilization (*Enterobacter* sp.) and used in Pb-containing soil for decontamination (Qu et al. 2022). The substrate enhanced P solubilization, leading to increased Pb removal due to the formation of insoluble Pb-P minerals.

The input of P in BC can also enhance soil microbial biomass (Turner and Wright 2014), while also altering soil microbial community composition and diversity (Yao et al. 2018; Ducousso-Détrez et al. 2022). It has been observed that P fertilization decreases the species richness of soil bacterial communities (Ling et al. 2017; Yao et al. 2018), and P accumulation may indirectly impact various ecological services by altering the microbial community (Beauregard et al. 2010). For instance, inorganic P addition has been found to reduce microbial secretion of phosphatase, thereby reducing the ability of soil microorganisms to solubilize organic P (Sinsabaugh et al. 2008; Marklein and Houlton 2012; Meng et al. 2024).

Another group of microorganisms employed in agriculture are Sulphur Oxidizing Bacteria (SOB), which oxidize elemental S into SO_4_^2−^, leading to soil acidification (Rezvani Boroujeni et al. 2021). This acidification can increase the solubility of P, consequently benefitting crop growth. SOB may be utilized in conjunction with BC, as studies have shown that BCs contain S at levels up to 1.5 mg g^−1^ (Zwetsloot et al. 2016; Wang et al. 2020). Amin and Mihoub (2021) tested BC from bovine bones with *Thiobacillus* spp. in solubilization experiments in calcareous soils, resulting in a significant increase in available P compared to control samples. However, further research is needed to assess the actual effects on plant growth. Overall, the combination of BC with microorganisms holds great potential for enhancing P solubilization, nutrient use efficiency, and sustainable fertilizer use (Fig. 3). Key knowledge gaps still remain in how BC affects the regulation and expression of key functional genes involved in microbial nutrient cycling and how different microbial species (i.e., within the bacterial, fungal and protozoal communities) and mesofauna respond to BC addition. Similarly, the effects of BC on root architecture, root hair density, AM colonization and P transporter expression remain unknown, even though these are known to be strongly regulated by exogenous P availability.

#### Environmental safety of BC application in agriculture

The production and application of BC require careful consideration of potential environmental impacts across its entire lifecycle. The manufacturing process involves high-temperature pyrolysis, which presents several environmental challenges. Without proper controls, the process could release GHGs, odours and particulate matter. Additionally, if pyrolysis temperatures are inadequately controlled, incomplete combustion can produce harmful byproducts including polycyclic aromatic hydrocarbons (PAHs) (Frišták et al. 2019; Pivato et al. 2024). The choice and sourcing of feedstock bones also requires consideration, as different sources may contain varying levels of contaminants (e.g., PTEs and pathogens) that affect final product quality.

Beyond production, the logistics chain presents its own environmental considerations. Collection and transportation of raw bone material requires biosecurity protocols to prevent pathogen spread (Cowie et al. 2012). Storage facilities must manage odour and leachate issues, while transport of both raw materials and finished BC needs to minimize dust generation and material losses. During field application, proper moisture management is crucial to prevent wind-blown losses and potential respiratory hazards for agricultural workers (Gwenzi et al. 2014; Gelardi et al. 2019). Worker safety protocols and appropriate personal protective equipment (PPE) are essential throughout the handling chain (Schwab and Hanna 2019).

Additionally, the long-term ecological impacts of BC application require further investigation. Key areas of concern include potential over-accumulation of P and trace elements in soil, raising the pH beyond the crop optima and effects on soil microbial community and soil function (El-Naggar et al. 2019). Additionally, while BC can sequester C, the net C footprint of production and application needs comprehensive life-cycle assessment across different production scales and technologies (Ramanan et al. 2022). The economic viability of different scales of production and their associated environmental impacts needs to be balanced against alternative P sources and soil amendments.

### Research gaps and future research on carbon sequestration

There are several key research gaps related to the application of BC in agriculture that need to be addressed to better understand its potential and limitations. One significant area is environmental safety. While BC has shown promise for contaminant remediation and nutrient delivery, there is a lack of long-term studies on its environmental impacts, particularly at the field scale. Specifically, more research is needed to assess the potential accumulation and release of PTEs and other contaminants in the soil and groundwater over time. These risks need to be better understood to prevent unintended environmental degradation or human health hazards. Additionally, the long-term effects of BC on soil (biological, chemical, and physical properties) and plant health are not well documented. Research should focus on how BC influences soil properties, nutrient cycles, and plant growth over extended periods and repeated applications, as well as its long-term efficiency as a P fertilizer compared to conventional options. Furthermore, work should be performed to optimize pyrolysis conditions to balance pathogen removal and P retention.

Another critical research gap involves the potential contribution of BC to climate change mitigation, which remains insufficiently studied. While biochar has been explored for its C sequestration properties, more focused research is needed on how BC can reduce GHG and ammonia emissions from agricultural soils. Understanding how BC can be optimized for both nutrient retention and climate benefits is essential for promoting its sustainable use in agriculture. Addressing these gaps will require multidisciplinary studies that combine environmental science, agronomy, and ecology to clarify the benefits and drawbacks of BC application. This research will help inform guidelines and policies, ensuring BC can be used safely and effectively in contributing to both sustainable agriculture and environmental protection.

Field-scale optimization also requires rigorous cost–benefit analyses to support its use in agriculture under a range of soil types, climatic zones, and cropping systems, with particular attention to how particle size and pyrolysis temperature affect the outcome. We therefore recommend that new studies are undertaken to replicate conditions predicted to occur beyond 2030 (e.g., whether bone char can promote drought tolerance in different types of soils under different climatic conditions and how it will impact on soil quality, crop yields and GHG emissions). We also lack long-term performance data on BC, specifically how BC particles degrade over time, how binding capacity evolves, and how these changes affect soil structure and C sequestration potential. Environmental impact studies must also assess the potential for dust generation during application, potential runoff effects, groundwater interactions, and broader ecosystem services. These investigations need to be coupled with detailed economic analyses and life cycle assessment that balance processing costs, transport efficiency, application equipment requirements, and storage infrastructure needs. Multi-year field trials across diverse agricultural and climatic zones would be particularly valuable in addressing these research gaps, especially when combined with standardized monitoring protocols and economic assessments.

## Conclusion

The evidence synthesis conducted here shows that BC has significant potential to address several environmental challenges, particularly in soil fertility management, enhanced soil C storage, contaminant removal and water purification. Its dual capability for slow P release and contaminant sorption, alongside its low cost, and renewable sourcing from high volume waste streams, makes it particularly valuable for resource-constrained regions. The versatility of BC in both agricultural and water treatment applications directly supports circular economy principles by transforming ABP-derived bones into a multi-functional resource, directly supporting SDG 2 (Zero Hunger), SDG 6 (Clean Water and Sanitation), and SDG 12 (Responsible Consumption and Production) through waste valorization.

However, critical research gaps remain in optimizing BC’s agricultural applications. Long-term field studies are needed to understand BC’s effects on soil fertility, microbial communities, and crop productivity across different soil types and climatic conditions. Production parameters, including pyrolysis temperature and feedstock composition, require further optimization to enhance BC’s performance and consistency. Additionally, the development of standardized quality metrics and application guidelines would facilitate wider adoption in agricultural practices. Applications of BC similarly need more research from an agricultural perspective (e.g., addition to animal wastes to reduce GHG and NH_3_ emissions, water treatment). Research priorities include enhancing contaminant removal efficiency, developing improved regeneration methods, and addressing scale-up considerations for community-level implementation.

Implementation research must parallel these technical investigations. Economic feasibility studies across different regions, development of supportive policy frameworks, and integration strategies with existing agricultural practices are essential. This includes examining supply chain logistics, establishing quality control measures, and creating market mechanisms to support BC adoption by farmers. Such comprehensive research will be crucial for realizing BC’s full potential in sustainable resource management and environmental protection while ensuring its practical viability in diverse global contexts.

## Data Availability

The data that support the findings of this study are available upon reasonable request.
